# Navigating Congenital Nasal Obstruction: A Contemporary Surgical Paradigm

**DOI:** 10.7759/cureus.53852

**Published:** 2024-02-08

**Authors:** Dinie Tumaisuri, Farhana Arif, Hardip Gendeh, Saraiza Abu bakar, Mohamad Safwan Zainuddin

**Affiliations:** 1 Otolaryngology, Hospital Shah Alam, Shah Alam, MYS; 2 Otolaryngology, Universiti Kebangsaan Malaysia Medical Center, Shah Alam, MYS; 3 Otorhinolaryngology, Hospital Shah Alam Selangor, Shah Alam, MYS; 4 Otorhinolaryngology, Head and Neck Surgery, Universiti Kebangsaan Malaysia, Kuala Lumpur, MYS; 5 Otorhinolaryngology, Hospital Serdang, Serdang, MYS; 6 Radiology, Hospital Shah Alam, Shah Alam, MYS

**Keywords:** stent, surgical dilatation, nasal obstruction, newborn, pyriform aperture stenosis

## Abstract

An uncommon form of nasal airway obstruction in a newborn with respiratory distress manifestations that needs prompt surgical correction when medical therapy cannot address the problem adequately. In this case study, two newborns were diagnosed with congenital nasal pyriform aperture stenosis (CNPAS) following a CT paranasal sinuses when the infant demonstrated persistent symptoms of upper airway obstruction. The narrowing of the nasal pyriform aperture, with a mean width of 0.65 cm in these newborns, was insufficient to allow breathing through the nostrils. Bedsides flexible endoscopy examinations revealed laryngomalacia in both of these infants. A supraglottoplasty, surgical nasal dilation, and stenting were performed without requiring a sublabial drill out of the pyriform aperture, allowing total resolution of the initial respiratory symptoms. Thus, a successful nasal enlargement was accomplished. During the post-operative follow-up period, no incurrences were observed.

Both patients with CNPAS were successfully treated with nasal dilatation and nasal stenting instead of the traditional pyriform aperture bone removal by a sublabial approach. Despite being a small series, it demonstrates that nasal dilatation and stenting may be considered an alternate procedure in selective CNPAS cases because it lowers the risk of open surgery and presumably offers an effective management option.

## Introduction

Congenital nasal pyriform aperture stenosis (CNPAS) is a rare cause of upper airway obstruction leading to respiratory distress in neonates. It was discovered for the first time by Brown in 1989 [1]. The cause is unknown, but it appears to be due to narrowing without occlusion of the anterior nasal bony aperture bounded superiorly by the nasal bones, the horizontal process of the maxilla and the anterior nasal spine inferiorly, and laterally by the nasal process of the maxilla [2].

CNPAS can exist in isolated form or be associated with other malformations such as holoprosencephaly, pituitary dysfunction, and a solitary median central incisor [3]. It manifests as apnea, cyclic cyanosis, difficulty in feeding, and abrupt total airway obstruction immediately after delivery, as the neonate is considered an obligate nasal breather until the eighth week of life, through which crying provides relief [4]. Physical examination with nasal endoscopy and CT sections must confirm the diagnosis in that the nasal pyriform aperture smaller than 11 mm is indicative of the diagnosis [5]. Corrective surgery is indicated in patients who fail to respond to medical therapy, present apnea or cyanosis symptoms, and lack the ability to make physical growth progress.

Two patients with CNPAS are presented. The purpose of this report is to first highlight the significance of prompt diagnosis in avoiding life-threatening pulmonary complications. Second, nasal dilatation with stenting may be a preferable therapeutic choice as opposed to the sublabial drill-out approach.

## Case presentation

Case 1

A full-term male newborn weighing 2.34 kg was delivered to non-consanguineous parents via spontaneous vertex delivery with a good Apgar score. The antenatal period was uneventful. The neonate was breathing spontaneously after birth, but during the first 24 hours of life, he developed respiratory distress, and chest recession with feeding difficulties requiring an endotracheal tube (ETT) size of 3.5 mm. He was immediately transferred to NICU (neonatal intensive care unit) for further pediatric evaluation and ventilation support. Chest radiography and laboratory workup upon NICU admission were both normal.

He was extubated after nine hours but was later reintubated due to worsening respiratory distress, tachypneic, and subcostal recess. Post-extubation for three days demonstrated that the child had noisy breathing, tachypnea with a moderate intercostal recession, and soft monophasic inspiratory stridor. Clinical examination of the child did not show any external dysmorphic features. There was no evidence of cleft palate.

**Figure 1:**
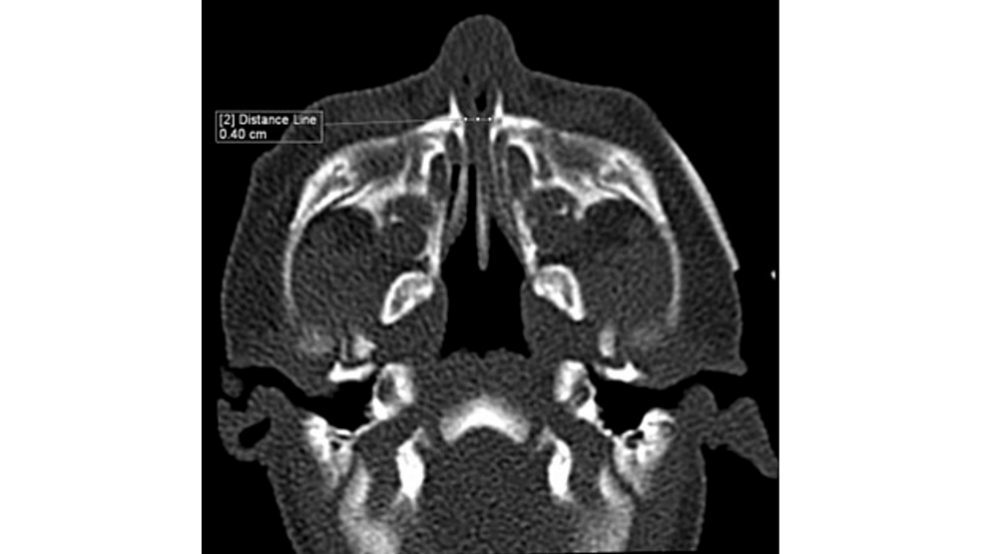
Axial CT scan shows the width of the pyriform aperture (4 mm).

The child was mouth-breathing. Nasal congestion was also apparent. Assessment of nasal patency by cold spatula test showed reduced misting from both nostrils. A size 6 French suction catheter was unable to pass through either nostril. Flexible endoscopy via oral cavity showed normal epiglottis but with tight AE folds, and the vocal folds were normal. The CT paranasal sinus scan revealed a narrow nasal pyriform aperture. The width of the pyriform aperture was 4 mm (Figure 1); hence, CNPAS was diagnosed as the cause of his dyspnea.

**Figure 2:**
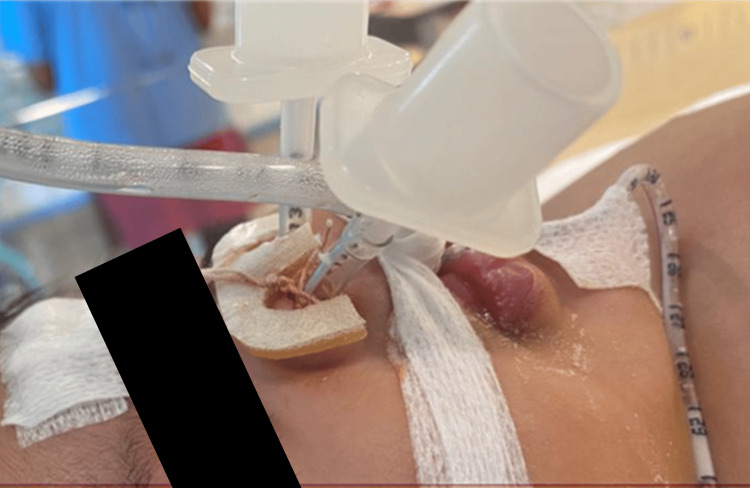
ETT size 3.0 stented in both nostrils to keep the patency. ETT, endotracheal tube

The patient, thereafter, underwent examination under anesthesia and nasal dilatation along with supraglottoplasty. Under general anesthesia, rigid laryngoscopy showed tight bilateral aryepiglottic folds and minimal redundant arytenoid mucosa with normal epiglottis and vocal fold. The nasal airways had improved with 3.0 endotracheal could easily be placed with no complications during and post-operative period (Figure 2).

Two days after the surgery, the patient successfully transitioned to normal breathing room air and shortly after began tolerating an oral diet. The nose was cleaned twice daily using nasal saline, decongestant nasal drops, intravenous antibiotic co-amoxiclav, and gentle suction. The stents were kept for six weeks, repeated nasal endoscopic examinations in the office at one-month post-procedure showed bilateral patent nasal passages, and the child remained asymptomatic.

Case 2

A 20-day-of-life female baby, born premature at 36 weeks, with a birth weight of 3.92 kg was delivered by cesarean section to a mother with a BMI of 34 and underlying type 2 diabetes mellitus. The first physical examination following birth was unremarkable with normal development and a good Apgar score. She was admitted to NICU after birth due to respiratory distress and was put on continuous positive airway pressure (CPAP) at 23 hours of life. Thus, she was referred to the ear, nose, and throat (ENT) team to rule out upper airway abnormalities. There were no external dysmorphic features but had intermittent noisy breathing with mild subcostal recession and inspiratory stridor upon vigorous crying.

There was no obvious mass seen in the nostrils and no evidence of cleft palate. There was reduced mist over the left nostril. A 6 French suction tube could not negotiate through the left nostril while the right was able to pass up to 8 French tube. The upper airway evaluation using a fiberoptic scope (FNPLS, flexible nasolaryngopharyngeal scope) with a diameter of 2.2 mm was performed through the oral cavity and showed floppy epiglottis, short and tight bilateral aryepiglottic fold, and redundant arytenoid mucosa, which were the features of laryngomalacia.

**Figure 3:**
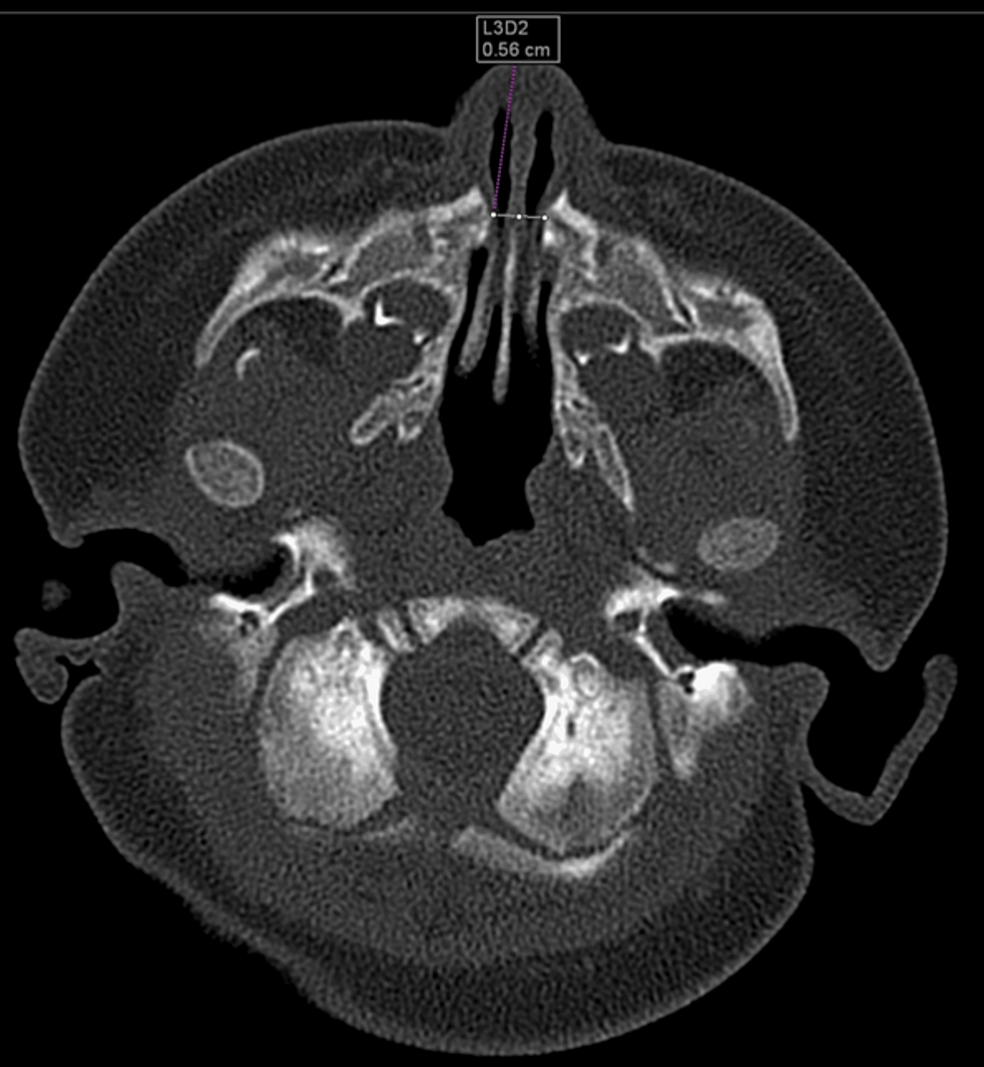
Axial section CT shows a narrow 5.6 mm diameter of pyriform aperture.

**Figure 4:**
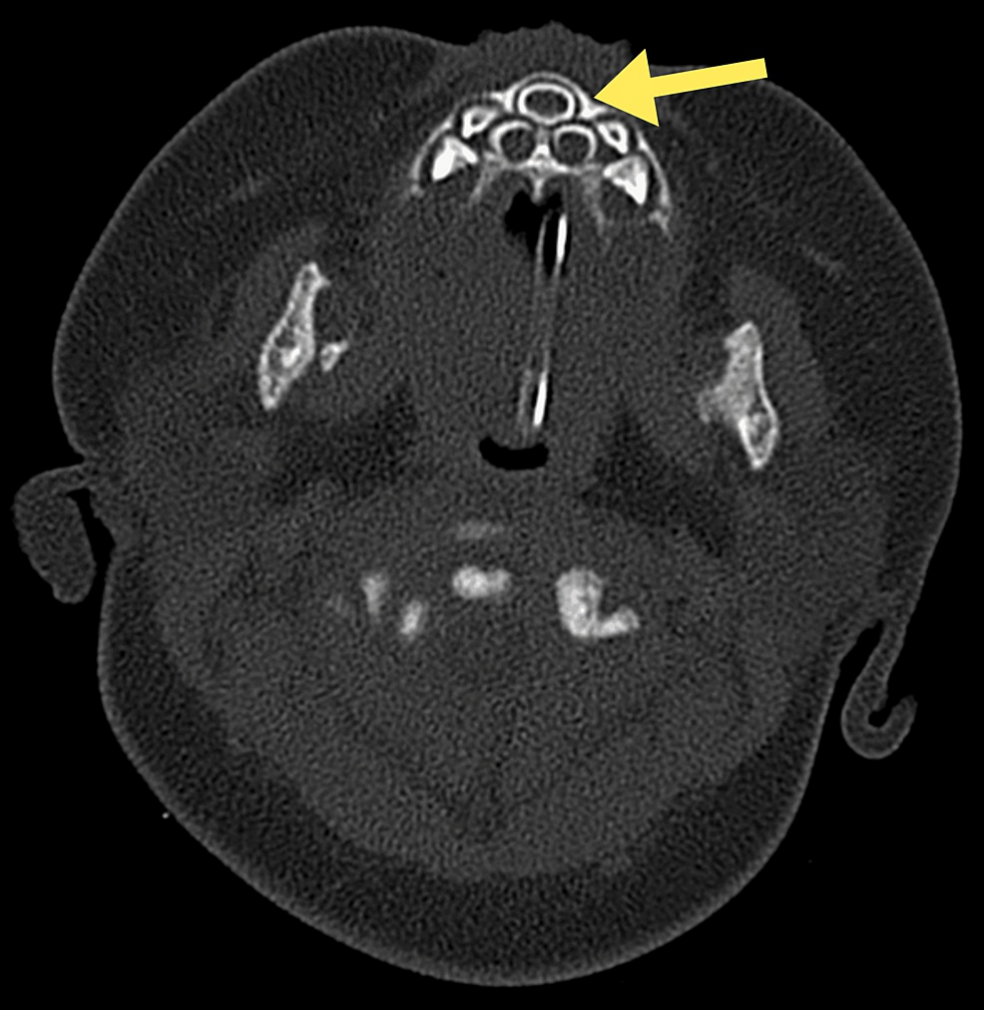
CT image shows SMMCI (yellow arrow). SMMCI, single median maxillary central incisor

**Figure 5:**
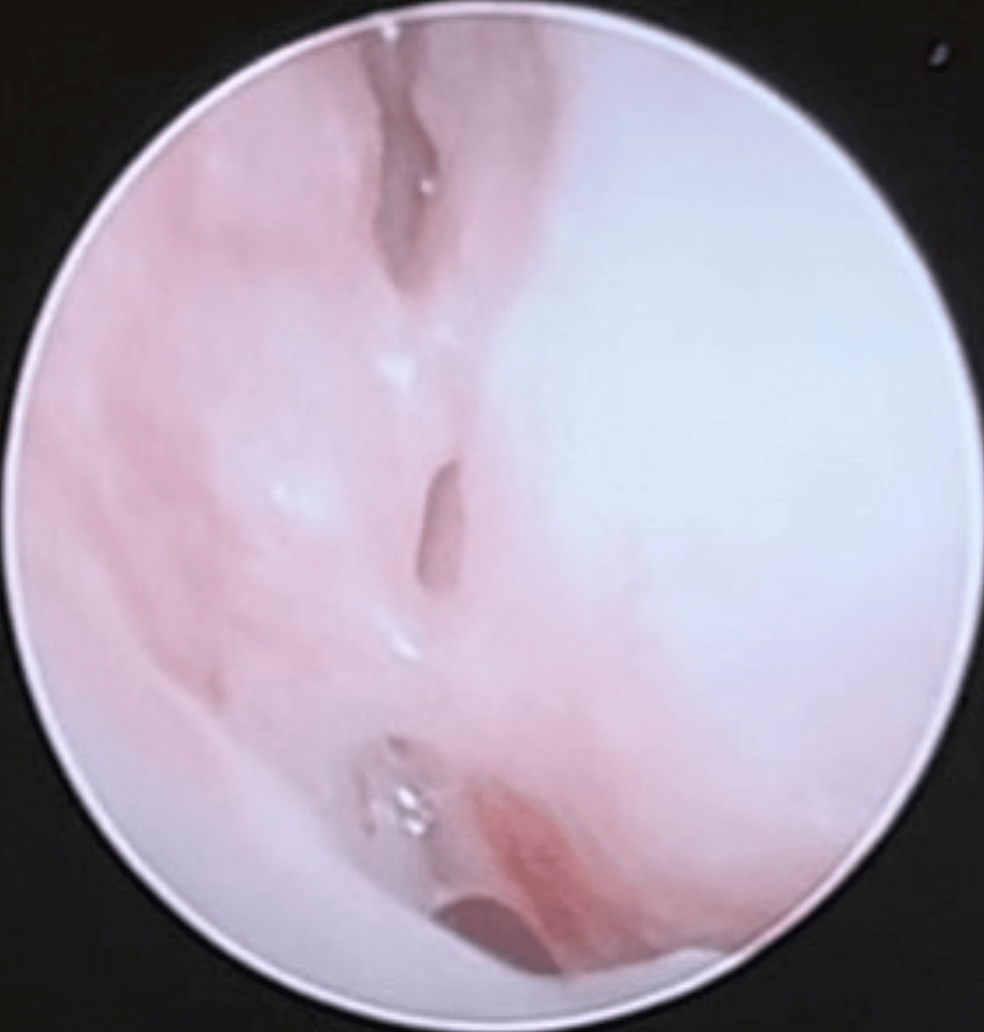
Rigid nasoendoscopy shows adhesion between the septum and right inferior turbinate.

**Figure 6:**
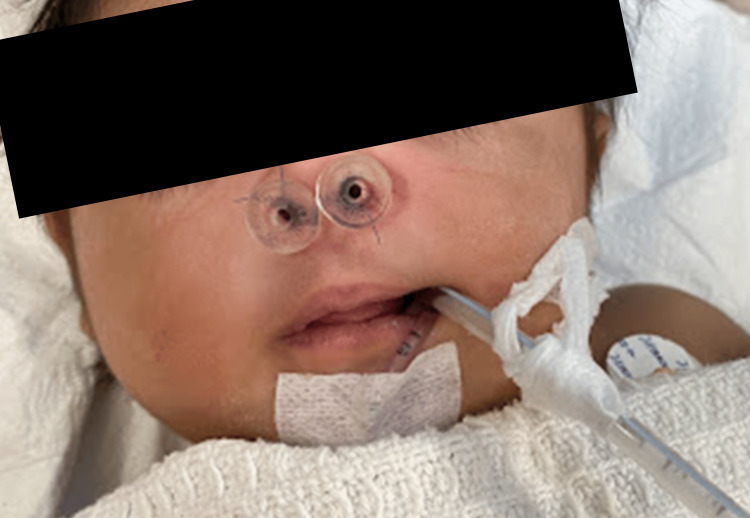
Bilateral nasal cavity stented using nasopharyngeal airway size 3.0.

Further imaging study through a non-contrast CT scan of the paranasal sinuses confirmed the diagnosis of pyriform sinus stenosis, which measured 5.9 mm in width on an axial image and single median maxillary central incisor (SMMCI) (Figure 3 and Figure 4). At 62 days of life, the child underwent surgery to dilate the stenosis and treat the laryngomalacia under general anesthesia. Under direct visualization, adhesiolysis was performed to remove the adhesion between the septum and bilateral inferior turbinate (Figure 5). Both the nasal cavities were dilated using ETT of size 3.0, and a nasopharyngeal airway device (NPA) was used as a stent and was fixed to the vestibules using nylon 4/0 (Figure 6). The position of the stents was confirmed endoscopically via the oral cavity. The patient tolerated the procedure well and was extubated on the table.

Both the stents were removed after six weeks. The patient was able to maintain saturation of oxygen and had a good air blast from both nasal cavities. Upon her follow-up appointment three weeks post-discharge, the patient showed overall well-being. There was no evidence of restenosis, and the baby continued to remain asymptomatic.

## Discussion

Because neonates are obligate nasal breathers, a slight reduction in the cross-sectional area at the pyriform aperture can result in significant increases in nasal airway resistance and lead to life-threatening respiratory distress. Common causes of bilateral nasal blockage tend to involve soft-tissue edema; therefore, the differential diagnosis includes nasal obstructions such as meningocele, meningoencephalocele, dermoid and epidermoid cysts, sinonasal tumors of any origin, septal dislocation or hematoma, nasal hypoplasia, choanal atresia, and CNPAS [4]. The incidence of CNPAS is predicted to be one in every 25,000 newborns and occurs at a frequency of about one-fourth to one-third of choanal atresia [4]. As a result, it is regarded as a rare clinical entity.

Anatomically, the nasal pyriform aperture is the narrowest and most anterior orifice of the bony nasal airway. It is a pear-like-shaped opening bounded superiorly by the nasal bone, inferiorly by the palatine process of the maxilla, and laterally by the nasal process of the maxilla. The etiology of the pathology is uncertain; however, it appears to be due to maxillary nasal process enlargement in the fourth month of fetal development [6].

It may occur alone or with other congenital anomalies such as holoprosencephaly, cleft palate, SMMCI, and central nervous system and endocrine problems [6]. 14-66% of CNPAS children have SMMCI [6]. Both posterior choanal atresia and CNPAS cause nasal airway obstruction and cyclical cyanosis that improves with crying and worsens with feeding. CNPAS symptoms may not appear until weeks after delivery, unlike bilateral choanal atresia, which is presented at birth. Delaying therapy puts the newborn at risk of respiratory distress and apnea, which can lead to ischemic brain damage and death. Failure of the suction tube to enter through the nostrils may indicate pyriform aperture stenosis [3].

As in our clinical case, the newborns were born with no craniofacial dysmorphism upon physical examination but immediate respiratory symptoms were observed [2]. When the pediatric team failed to wean the child off from oxygen for more than a month and a 2.5 mm pediatric flexible endoscope with the nasal suction tube could not be advanced through either nasal vestibule, diagnosis of CNPAS was considered. Although it is a very rare cause of neonatal airway obstruction, the diagnosis of CNPAS in this newborn was slightly delayed [6]. The possibility of CNPAS arose when the pediatric team was unable to wean the child off of oxygen. The diagnosis was slightly delayed in view of the fact that CNPAS is a rare cause of neonatal airway blockage.

There are currently no reliable criteria for determining whether CNPAS warrants surgical treatment. Lin et al. propose that not all symptomatic CNPAS cases necessarily require surgery [6]. Indications for surgery include failure to thrive, dependence on supplemental oxygen, obstructive sleep apnea, and cyanosis [7]. Surgical intervention is generally recommended when conservative treatments prove ineffective.

The correction method involves a sublabial approach, overgrown bone removal, and stenting, which is a standard procedure but carries a reported 14% failure rate [7]. This approach has limitations, including the risk of lacrimal system and dental bud injury due to their proximity to the surgical site. Post-operatively, stenting, which is often with endotracheal tubes, has historically been employed, despite possible complications [7].

The primary advantage of stenting is that it is less invasive, but multiple dilations appear to be necessary [7]. The main area of interest of these stents is the mucosa. In the context of CNPAS, the use of these stents demonstrates the potential for enhanced treatment with fewer side effects. Approximately six weeks after the procedure, our patient was able to be extubated and tolerated an oral diet on the day of extubation (Table 1) [7].

**Table 1:** Comparison of surgical approach in patients with CNPAS CNPAS, congenital nasal pyriform aperture stenosis; ETT, endotracheal tube

Surgery	Indication	Procedure performed
Sublabial drill out	Pyriform aperture width less than 11 mm associated with median central incisor	Enlargement of the pyriform aperture using a powered drill
Nasal stenting	Mucosa of the inferior turbinates at the lateral border of the pyriform aperture	Gradual dilation using bougie from size 10Fr, 12Fr, and 14Fr and stenting with ETT size 3.0 mm

There are multiple advantages to avoiding the sublabial drill out. Typically, 1-2 mm of bone is removed from the lateral wall of the pyriform aperture. As an outcome, there is a risk of harm to the nasal mucosa, nasolacrimal duct, tooth buds, nasal ala, and septum [8]. We regarded these patients as successful despite the delayed approach since stenting and dilatation allowed normal respirations, oral nourishment, weight growth, and the avoidance of sublabial drill out [8]. Ultimately, additional patients have to be evaluated to determine whether this procedure has a lower recurrence rate than standard stenting.

Nasal stenting is recommended to be used to provide a patent nasal airway, immediate relief of the nasal obstruction while the operative site is healing, and also to prevent recurrence and scar-related stenosis [6]. The length of the nasal stent should be based on the length of the nasal stenosis where concomitant pathology should be taken into account. In isolated CNPAS, a shorter stent should be applied while a longer nasal stent should be used in patients with choanal atresia to prevent obstructive scarring in the posterior nasal area [6]. We regarded these patients as successful despite the delayed approach since dilatation allowed normal respirations, oral nourishment, weight growth, and the avoidance of sublabial drill out [8]. Post-operative nasal saline and suctioning avoid crusting and stent blockage, which could be life-threatening. If the child is transferred to the nearest health center with a stent, parents must be reassured to suction with saline drops [9].

Our experience implies that nasal airway dilation reduces symptoms and improves outpatient medical treatment after discharges. The length of hospitalization following nasal dilatation varied from 10 to 14 days, whereas a series of surgical conventional drill-out procedures reported a range of 10 to 31 days [8]. Thus the overall cost of treating CNPAS patients can be minimized by reducing inpatient medical costs and the amount of work days missed by the parents. Therefore, nasal dilatation is a simple, less invasive procedure that could become capable of providing symptomatic relief with improved health care usage. It may complement medical treatment and minimize the requirement for the standard sublabial pyriform aperture drill out.

This investigation is limited by the absence of a control group and another experimental group. A control group would have been useful for comparing the results of groups that received only medical therapy. In our series, however, both neonates were treated with dilation due to the failure of medical therapy. Moreover, a comparison with an experimental group that received a pyriform aperture drill out would have been beneficial. Due to the rarity of this disease, an optimal investigation with multiple cohorts is impractical. Infrequent occurrences also restricted the sample size of this first report of a novel therapeutic method.

## Conclusions

Symptomatic improvement in these two consecutive CNPAS patients with feeding and respiratory difficulties suggests that nasal dilation may be an effective treatment option for CNPAS. CNPAS is a differential diagnosis and early detection of the diagnosis and early intervention are important for effective results.

The effective utilization of nasal dilatation and stenting as a substitute for conventional surgical methods indicates a hopeful alternative, diminishing the potential hazards linked to invasive surgery. This suggests that a minimally invasive approach could provide effective treatment, demonstrating the possibility of wider use in specific cases of CNPAS. Additional studies and extensive studies could yield useful insights regarding the feasibility and applicability of this strategy.
